# STACKing the odds for adolescent survival: health service factors associated with full retention in care and adherence amongst adolescents living with HIV in South Africa

**DOI:** 10.1002/jia2.25176

**Published:** 2018-09-21

**Authors:** Lucie Cluver, Marija Pantelic, Elona Toska, Mark Orkin, Marisa Casale, Nontuthuzelo Bungane, Lorraine Sherr

**Affiliations:** ^1^ Department of Social Policy and Intervention University of Oxford Oxford United Kingdom; ^2^ Department of Psychiatry and Mental Health University of Cape Town Cape Town South Africa; ^3^ Secretariat International HIV/AIDS Alliance Brighton United Kingdom; ^4^ AIDS and Society Research Unit University of Cape Town Cape Town South Africa; ^5^ Department of Sociology University of Cape Town Cape Town South Africa; ^6^ MRC/Wits Developmental Pathways for Health Research Unit School of Clinical Medicine University of the Witwatersrand Johannesburg South Africa; ^7^ School of Public Health University of the Western Cape Cape Town South Africa; ^8^ Department of Nursing Fort Hare University Alice South Africa; ^9^ Research Department of Global Health University College London London United Kingdom

**Keywords:** adolescent, HIV, delivery of healthcare, medication therapy management, adolescent health services, viral load

## Abstract

**Introduction:**

There are two million HIV‐positive adolescents in southern Africa, and this group has low retention in care and high mortality. There is almost no evidence to identify which healthcare factors can improve adolescent self‐reported retention. This study examines factors associated with retention amongst antiretroviral therapy (ART)‐initiated adolescents in South Africa.

**Methods:**

We collected clinical records and detailed standardized interviews (n = 1059) with all 10‐ to 19 year‐olds ever initiated on ART in all 53 government clinics of a health subdistrict, and community traced to include lost‐to‐follow‐up (90.1% of eligible adolescents interviewed). Associations between full self‐reported retention in care (no past‐year missed appointments and 85% past‐week adherence) and health service factors were tested simultaneously in sequential multivariate regression and marginal effects modelling, controlling for covariates of age, gender, urban/rural location, formal/informal housing, maternal and paternal orphanhood, vertical/horizontal HIV infection, overall health, length of time on ART and type of healthcare facility.

**Results:**

About 56% of adolescents had self‐reported retention in care, validated against lower detectable viral load (AOR: 0.63, CI: 0.45 to 0.87, *p* = 0.005). Independent of covariates, five factors (STACK) were associated with improved retention: clinics Stocked with medication (OR: 3.0, CI: 1.6 to 5.5); staff with Time for adolescents (OR: 2.7, CI: 1.8 to 4.1); adolescents Accompanied to the clinic (OR: 2.3, CI: 1.5 to 3.6); enough Cash to get to clinic safely (OR: 1.4, CI: 1.1 to 1.9); and staff who are Kind (OR: 2.6, CI: 1.8 to 3.6). With none of these factors, 3.3% of adolescents reported retention. With all five factors, 69.5% reported retention.

**Conclusions:**

This study identifies key intervention points for adolescent retention in HIV care. A basic package of clinic and community services has the potential to STACK the odds for health and survival for HIV‐positive adolescents.

## 1 Introduction

Two million HIV‐positive adolescents (aged 10 to 19) live in Sub‐Saharan Africa, both horizontally and vertically infected. This age group has the lowest rates of retention in HIV care, and the lowest adherence to antiretroviral therapy (ART) 1. Consequently, adolescents have elevated risks of viral failure, morbidity and mortality, and onwards HIV transmission risk 2. HIV/AIDS‐related deaths amongst adolescents have tripled since 2000, with AIDS now the leading cause of death amongst adolescents in the region 3.

Studies show barriers to adolescent use of HIV care and challenges in transitioning from paediatric to generalized adult services 4. A recent situational analysis of 218 ART‐providing health facilities across Sub‐Saharan Africa found very low provider knowledge of the specific needs of adolescents in HIV care 5. Evidence suggests that services that improve retention amongst HIV‐positive adults, such as support groups, may be less effective for adolescents 6. In response, there are increasing calls and efforts to create adolescent‐responsive health systems (e.g. adolescent‐friendly clinics, peer support programmes), particularly in Sub‐Saharan Africa 7.

However, there is a lack of quantitative evidence to guide the specific content of adolescent‐responsive and enabling services in the global South 8. Two systematic reviews identify potential impacts in high‐income settings of weekend treatment breaks 9, psychosocial interventions, observed therapy, financial incentives and extended clinic opening hours, but noted very small sample sizes in existing studies 10, 11. A US‐based observational study found higher youth retention in clinics with youth‐friendly waiting areas, evening clinic hours and providers trained in adolescent care 12. In Haiti, a pre/post study of an adolescent‐friendly clinic showed improved ART initiation but no differences in retention 13. In the African region, qualitative studies with young people suggest perceived value of youth groups, supportive healthcare staff and financial assistance for transport to clinics 14, 15. Systematic reviews of health services that predict adult retention in HIV care in low‐resource settings have identified factors of ART counselling at initiation, lower staff workload in the clinic, community‐based service delivery, down‐referral of stable patients and differentiated care. It is noted, however, that none of these factors had been shown to be effective amongst adolescents 16, 17.

There is a clear need to identify modifiable health service factors associated with adolescent retention in care in Sub‐Saharan Africa. This study aims to contribute to this evidence base by asking (1) what modifiable health service factors are associated with full retention in HIV care amongst HIV‐positive adolescents and (2) can combinations of factors have additive promotive effects, in order to identify an effective minimum basket of provisions.

## 2 Methods

In this cross‐sectional study, interviews and clinical records were collected from HIV‐positive adolescents in South Africa. Recruitment took place from March 2014 to September 2015. The study site was a rural, peri‐urban and urban health subdistrict in the Eastern Cape province, an area where the healthcare system experiences high burden, poor infrastructure and human resource challenges 18. All health facilities that provided ART to 5 or more adolescents were included (n = 53, including hospital antenatal, paediatric and ART clinics, community health centres and primary care clinics). In each health facility, all clinical files (paper and computerized) were reviewed to identify all adolescents aged 10 to 19 who had ever initiated ART, irrespective of current or past health service attendance. In order to ensure inclusion of adolescents who were both attending and not attending clinical care (and to avoid selection bias of only including those retained in care), all adolescents identified in these files were traced to 180 communities and interviewed at home.

Ethical approval was given by the University of Cape Town (CSSR 2013/4) and Oxford University (SSD/CUREC2/12‐21), as well as the Provincial Departments of Health and Education and participating health facilities. All adolescents and their primary caregivers gave written informed consent for participation, and consent procedures were also read aloud in case of low literacy. No financial incentives were given, but all adolescents received a snack, small gift pack (selected by the project's Teen Advisory Group and including soap, deodorant and pencils) and a certificate. To prevent these becoming an incentive, adolescents received packs and certificates regardless of whether they consented to participate in the study. In order to prevent stigma or unwanted disclosure, the certificate (and all study materials) did not refer to HIV or AIDS but instead to a study about general health and social needs of adolescents in South Africa. Confidentiality was maintained except in cases of risk of harm: where participants reported abuse, suicidality, rape or severe untreated illness, referrals were made to relevant health or social services (n = 94 referrals in the full sample), and followed up to ensure that services were received.

Participants completed tablet‐based questionnaires lasting 60 to 90 minutes, with the support of researchers trained in working with vulnerable adolescents. Questionnaires were designed with adolescents (the study's Teen Advisory Group) to be engaging and non‐stigmatizing, and were piloted with 25 HIV‐positive adolescents in the Eastern Cape. Measures were translated and back‐translated into the local language (Xhosa) and were completed in the language of participants’ choice. In order to identify potential health service factors that were relevant and modifiable, we collaborated with the South African National Departments of Health, Social Development and Basic Education, the South African National AIDS Council, UNICEF, PEPFAR‐USAID and NGOs including Pediatric Adolescent Treatment for Africa.

### 2.1 Measures

Full questionnaires are available at http://www.mzantsiwakho.co.za. *Full self‐reported retention in care* was defined as a combination of attending clinic appointments and adhering to ART, defined as both no missed clinic visits over the past year and 85% adherence over the past week, following WHO recommendations 19. Missed appointments were measured over the past year, and used patient self‐report due to low rates of recording of appointments in patient files, low availability of files to healthcare providers when seeing patients and high rates of adolescent mobility between clinics. ART adherence was measured over the past week in order to minimize recall bias, and to include weekdays and weekend given literature on weekend variation. Adherence items used the standardized Patient Medication Adherence Questionnaire 20, adapted using measures developed in Botswana 4. A validation measure was taken in order to test the reliability of self‐reported retention in care: a *detectable viral load* was extracted from clinical records and defined as viral load 50 + /ml 21.

In total, 11 potential protective health service factors were measured, all using adolescent self‐report. Factors hypothesized to increase access to the clinic were (all reported for the past year): (1) less than one hour travel to the clinic from the adolescent's home; (2) the clinic is accessible: the adolescent can afford to get to the clinic and feels safe whilst travelling and entering the clinic; (3) the adolescent is accompanied to the clinic (either by someone from home or by clinic support staff); and (4) waiting time at the clinic is less than one hour. Factors hypothesized to improve healthcare experience and ART adherence were: (5) the clinic has a reliable antiretroviral stock (i.e. no stock outs in the past year); (6) the clinic healthcare providers have enough time to talk to adolescents; (7) the adolescent perceives that the clinic healthcare providers are kind to adolescents; (8) the clinic healthcare staff provide adolescents with the information they request; (9) the adolescent feels as though their personal information would be kept confidential; (10) the adolescent attends a regular support group that meets at least monthly and; (11) the adolescent has an identified treatment buddy. All measures were dichotomized.

In total, 10 potential covariates were measured and controlled for in all analyses, using adolescent self‐report and clinical records: (1) age (dichotomized as younger adolescents aged 10 to 14 and older adolescents aged 15 to 19); (2) gender; (3) residential location (urban/rural); (4) housing situation (formal/informal) were measured using items based on South Africa's Census 22; (5) maternal orphanhood and (6) paternal orphanhood were measured using items recommended by UNICEF 23; (7) vertical/horizontal HIV infection was assessed following existing Sub‐Saharan African paediatric HIV cohorts: an age cutoff for initiation (10 years) 24 was validated with a detailed algorithm which evaluated the consistency of the initial designations, with inconsistent designations being recoded when strong evidence was available (i.e. maternal and paternal death); (8) overall adolescent health was self‐reported over the past 6 months using the WHO ICF checklist 25; (9) length of time on ART treatment was measured via self‐report and clinic records (more/less than one year on treatment); (10) type of healthcare facility was recorded by the research team and dichotomized into paediatric care versus adult (primary care, adult or antenatal care). We also measured the level of health facility that is primary (clinics), secondary (community health centres/day hospitals) and tertiary care (hospital). Given that access to tertiary care was highly correlated with paediatric care (0.704, *p* < 0.001), this study only controlled for access to paediatric care.

### 2.2 Analysis strategy

**Figure 1:**
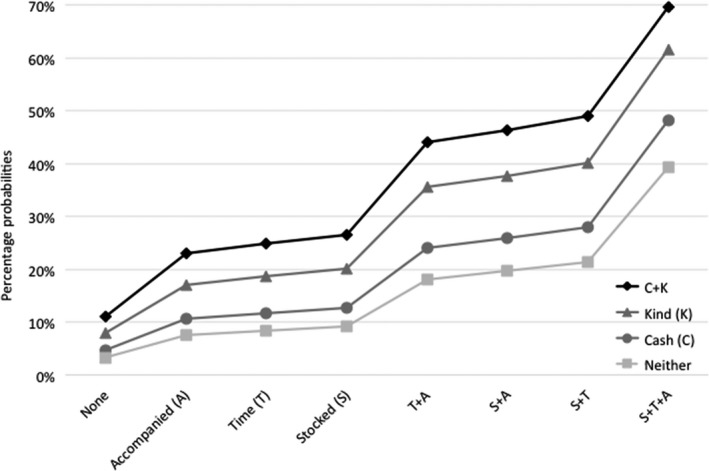
Percentage probabilities of retention in care (controlling for covariates).

Analyses were conducted in five stages in SPSS 22 and STATA 14. The first three stages were to check the reliability of the sample and outcome measure, and assess frequencies. The final two stages were to identify factors associated with self‐reported retention in care and potential cumulative associations of combinations of those factors. First, eligible participants included in the study were compared to those excluded (the 9.9% not traceable or refused participation) on the sociodemographic characteristics that were available for both groups (age, gender, urban/rural location) using chi‐square tests. Second, frequency distributions for all outcomes, potential protective provisions and covariates were reported. Third, associations of self‐reported retention in care were tested in multivariate logistic regressions, against a validation measure of detectable viral load, controlling for potential covariates (Table 3). Fourth, we ran sequential logistic regressions following Hosmer and Lemeshow's recommendations 26 to test potential associations between individual clinic‐level protective factors and adolescent retention in care. The first step was a model including all potential covariates and health service factors; the second model retained covariates and health service factors significant at *p *<* *0.1; the third and final model retained covariates and health service factors significant at *p *<* *0.05 (Table 4). In the fifth stage, to test potential cumulative effects of protective health service factors, a marginal effects model was run with all potential combinations of significant protective factors, holding significant sociodemographic and HIV‐related cofactors at mean values (Figure 1). This was plotted with 95% confidence intervals.

## 3 Results

Of the 1176 eligible participants, 90.1% (n = 1059) were included. About 4.1% of the adolescents or their caregivers refused participation, 0.9% were unable to participate because of severe cognitive delay, 3.7% were not traceable and 1.2% no longer lived in the study area. An additional 20 participants were recorded as living in clinic files, but when researchers visited their homes were identified to have died. In order to avoid risk of stigma, the study was presented in communities as focused on general adolescent health and social service use, and an additional 467 adolescents were interviewed who were coresident or living in neighbouring homes (not included in analyses).

Stage 1: No significant differences on age, gender or urban/rural location were found between the interviewed and non‐interviewed samples (Table 1). Stage 2: The sample was 55% female, with a mean age of 13.8. 22% lived in a rural area. Just under half of adolescents were maternal orphans (44%) and 30% paternal orphans (Table 2). Three quarters were vertically infected, and 47% received care in a paediatric clinic. Access to hypothesized protective healthcare factors ranged from high (i.e. 88% reported that clinic staff had enough time to spend with them, 94% of clinics had no stock outs in the past year) to low (i.e. 14% attended a monthly support group). About 56% of adolescents reported full retention in HIV care that is both no missed clinic visits over the past year (84%) and full adherence over the past week (64%).

**Table 1:** Comparisons between reached and unreached adolescents

	HIV positive (n = 1059)	Excluded (n = 116)	Comparison tests
Age (mean, SD)	13.8, 2.834	14.8, 2.91	*p* = 0.671
Female (n, %)	587, 55.2%	66, 56.9%	*p* = 0.769
Rural (n, %)	228, 21.4%	26, 22.4%	*p* = 0.813

*p* values associated with z score and chi‐square tests.

**Table 2:** Frequencies (n = 1059)

	n, %
Outcome	
Full retention	591, 55.8
Full clinic attendance (past year)	885, 83.5
Full adherence (past week)	764, 63.6
Undetectable VL (<50 copies/ml) (n = 702)	238, 32.5
Sociodemographic and HIV potential covariates	
Age (10 to 14)	659, 62.2
Female gender	584, 55.1
Rural household location	228, 21.5
Informal housing	198, 18.7
Maternal orphan	460, 43.7
Paternal orphan	318, 30.2
Horizontally acquired HIV	269, 25.4
Good overall health	430, 40.8
>1 year on ART	752, 70.9
Paediatric clinic care	499, 47.1
Health service factors	
Travel time to clinic <1 hour	940, 88.7
Clinic accessible	738, 69.6
Feels safe going to clinic	930, 87.7
Enough cash to get to clinic	819, 77.3
Accompanied to clinic	930, 87.7
Clinic waiting time <1 hour	368, 34.7
Clinic well stocked with ART	1001, 94.4
Clinic staff have enough time	931, 87.8
Clinic staff are kind	858, 80.9
Clinic staff provide information	946, 89.2
Perceived confidentiality at clinic	804, 75.8
Monthly support group	144, 13.6
Treatment buddy	757, 71.4

Stage 3: Limited health service capacity in the province meant that viral load testing was not consistently performed or recorded: 704 adolescents (66.4%) had a viral load recorded in their patient files within the past two years. In this group, self‐reported higher retention in care was significantly negatively associated with detectable viral load (AOR: 0.63, CI: 0.45 to 0.87, *p *=* *0.005) independent of age, gender, urban/rural location, formal/informal housing, maternal orphanhood, paternal orphanhood, mode of infection, time on ART treatment and travel time to clinic. Hosmer and Lemeshow tests indicated good model fits for the validation against detectable VL (χ^2^ (df) = 6.54 (8), *p *=* *0.587) (Table 3).

**Table 3:** Multivariate logistic regression analyses testing associations between self‐reported full retention in care and detectable viral load

	Detectable VL (n = 704 biological markers)	
	AOR	95% CI
Potential covariates		
Age (10 to 14)	0.699	0.466 to 1.049
Female gender	0.944	0.681 to 1.308
Rural household location	1.179	0.792 to 1.757
Informal housing	1.374	0.913 to 2.066
Maternal orphan	1.436*	1.027 to 2.007
Paternal orphan	1.141	0.797 to 1.633
Horizontally acquired HIV	1.139	0.727 to 1.786
Good overall health	0.853	0.612 to 2.040
>1 year on ART	0.610	0.287 to 1.296
Paediatric clinic care	0.745	0.519 to 1.069
Outcome measure		
Full retention (self‐reported)	0.629**	0.453 to 0.874

**indicates *p *<* *0.005; *indicates *p *<* *0.05.

In Stage 4, we tested associations of all potential protective factors simultaneously, controlling for all potential covariates, with adolescent self‐reported retention in care (Table 4). In the first model, all the 10 potential protective factors were included. Five factors did not meet the *p *<* *0.1 cutoff and were therefore excluded from step 2: clinic healthcare workers providing adolescents with the information they request; having a treatment buddy; attending a monthly support group; confidentiality of information and travel time to clinic. Based on step 2, waiting time at clinic was additionally excluded due to having a *p *>* *0.05. Of the remaining five health service factors, all were significant at *p* < 0.05 in the third and therefore final model. Controlling for all health service protective factors simultaneously and covariates significant at *p* < 0.05, the following factors were positively associated with adolescent retention in care: clinics that were fully stocked with medication (AOR: 3.0, CI: 1.6 to 5.5); staff with enough time for adolescents (AOR: 2.7, CI: 1.8 to 4.1); adolescents that were accompanied to the clinic (AOR: 2.3, CI: 1.5 to 3.6); having enough cash to get to clinic and safety on the way (AOR: 1.4, CI: 1.1 to 1.9); and staff who are perceived as kind to adolescents (AOR: 2.6, CI: 1.8 to 3.6). The Hosmer and Lemeshow test indicated that the final model fitted the data well (χ^2^ (df) = 2.851 (6), *p *=* *0.827). Correlation matrices found no risk of collinearity between independent variables.

**Table 4:** Results of the three‐step sequential model. Step 3 presents the final model results

	AOR	Lower CI	Upper CI
Step 1			
Potential covariates			
Age (10 to 14)	0.958	0.676	1.356
Female gender	0.934	0.705	1.238
Rural household location	0.724^	0.534	1.141
Informal housing	0.986	0.692	1.405
Maternal orphan	1.193	0.886	1.605
Paternal orphan	0.962	0.717	1.292
Horizontally acquired HIV	0.781	0.534	1.141
Good overall health	1.130	0.844	1.512
>1 year on ART	1.152	0.820	1.619
Paediatric clinic care	1.634**	1.200	2.224
Health service factors			
Travel to clinic <1 hour	1.393	0.908	2.136
Clinic accessible (adolescent can afford to get to the clinic and feels safe whilst travelling)	1.427*	1.048	1.944
Accompanied to clinic	2.439***	1.571	3.789
Waiting time at clinic <1 hour	0.750*	0.565	0.996
Clinic well stocked with ART	3.159***	1.692	5.900
Clinic staff have enough time	2.744***	1.762	4.274
Clinic staff are kind	2.731***	1.909	3.906
Clinic staff provide information	1.238	0.780	1.964
Perceived confidentiality at clinic	0.931	0.672	1.288
Monthly support group	1.205	0.794	1.826
Treatment buddy	0.795	0.570	1.108
Step 2			
Potential covariates			
Rural household location	0.723	0.520	1.006
Paediatric clinic care	1.770***	1.345	2.330
Health service factors			
Clinic accessible (adolescent can afford to get to the clinic and feels safe whilst travelling)	1.420*	1.052	1.916
Accompanied to clinic	2.425***	1.588	3.704
Waiting time at clinic <1 hour	0.822	0.628	1.075
Clinic well stocked with ART	3.019***	1.639	5.560
Clinic staff have enough time	2.748***	1.782	4.237
Clinic staff are kind	2.569***	1.815	3.637
Step 3 (final model)			
*Potential covariates*			
Paediatric clinic care	1.895***	1.449	2.479
Health service factors			
Clinic accessible (adolescent can afford to get to the clinic and feels safe whilst travelling)	1.423*	1.056	1.919
Accompanied to clinic	2.349***	1.543	3.578
Clinic well stocked with ART	3.016***	1.640	5.545
Clinic staff have enough time	2.671***	1.735	4.114
Clinic staff are kind	2.564***	1.814	3.625

***indicates *p *<* *0.001; **indicates *p *<* *0.005; *indicates *p *<* *0.05; ^indicates *p < *0.1 (in step 1).

In Stage 5, potential cumulative effects were tested in marginal effects models (see Figure 1), and showed a clearly graded pattern of increased rates of retention in HIV care associated with increased access to STACK factors (Table 5). Rates of full retention amongst adolescents with none of the protective STACK factors was 3.3%, rising to 4.7% and then to 9.2% with any single protective factor. With any two factors, retention in care ranged from 10.6% to 21.3%, with any three from 22.9% to 40.2%, and with any four from 44% to 61.5%. With all five STACK factors, full retention in care was 69.5%.

**Table 5:** Predicted probabilities of full retention by access to protective health service factors (STACK)

Protective health service factors	# factors	Likelihood of full retention	Error margin
None	0	3.3%	2.7%
Cash	1	4.7%	3.9%
Accompanied	1	7.6%	5.3%
Kind	1	7.9%	5.9%
Time	1	8.4%	5.8%
Stocked	1	9.2%	5.1%
Accompanied & Cash	2	10.6%	7.3%
Cash & Kind	2	11.0%	8.1%
Time & Cash	2	11.7%	8.0%
Stocked & Cash	2	12.7%	7.1%
Accompanied & Kind	2	17.1%	10.2%
Time & Accompanied	2	18.1%	9.9%
Time & Kind	2	18.7%	10.6%
Stocked & Accompanied	2	19.6%	7.9%
Stocked & Kind	2	20.2%	9.1%
Stocked & Time	2	21.3%	8.1%
Accompanied, Cash & Kind	3	22.9%	12.6%
Time, Accompanied & Cash	3	24.1%	12.1%
Time, Cash & Kind	3	24.8%	13.2%
Stocked, Accompanied & Cash	3	25.9%	9.5%
Cash, Kind & Stocked	3	26.6%	11.2%
Stocked, Time & Cash	3	28.0%	10.1%
Time, Accompanied & Kind	3	35.5%	14.0%
Stocked, Accompanied & Kind	3	37.7%	10.6%
Stocked, Time & Accompanied	3	39.3%	8.6%
Stocked, Time & Kind	3	40.2%	10.0%
Time, Accompanied, Cash & Kind	4	44.0%	14.5%
Stocked, Accompanied, Cash & Kind	4	46.3%	10.4%
Stocked, Time, Accompanied & Cash	4	48.2%	8.4%
Stocked, Time, Cash & Kind	4	49.0%	10.2%
Stocked, Time, Accompanied & Kind	4	61.5%	6.5%
STACK (All protective health service factors)	5	69.5%	3.7%

## 4 Discussion

Since 2000, there have been 5.7 million new adolescent HIV infections globally, combined with increasing numbers of children infected with HIV surviving into adolescence 27. About 20% of all HIV‐positive adolescents live in South Africa 24. It has become clear that they have unique challenges in engaging with chronic antiretroviral use and HIV services 28, and require targeted responses in order to reach acceptable levels of treatment retention. Individualized care (with specialized care plans and providers for each patient) has been linked to high adolescent retention 29, but may not be feasible in national‐scale government health services in Sub‐Saharan Africa.

This paper identifies five protective health service factors associated with self‐reported full retention in care amongst HIV‐positive adolescents in South Africa. All factors tested were those already occurring (to varying extents) within existing services for adolescents, and this analysis therefore provides evidence of potentially feasible and affordable provisions within government services in Southern Africa. These factors summed into the acronym STACK: clinics fully Stocked with medication; staff with enough Time for adolescents; adolescents that were Accompanied to the clinic; having enough Cash to get to clinic and safety on the way; and staff who are perceived to be Kind to adolescents.

Two factors were related to reaching the clinic. Unexpectedly, travel time and waiting time at the clinic were not significantly associated with retention when controlling for other factors, but having enough money and safe access to the clinic (either on foot or through public transport) were associated with increased retention. In addition, being accompanied to the clinic was associated with more than double the odds of retention. These suggest potential value of relatively low‐cost interventions at the household and/or clinic level. For example, some of the included clinics had Kheth'Impilo patient advocates (lay community healthcare workers) who accompanied adolescents to services when their families could not 30. The provision of transport vouchers was shown to increase adult retention in care in Uganda 31.

The reliability of treatment supplies in the clinic was associated with threefold odds of increased retention in care. This reflects similar findings in adult populations 32. Health systems face multiple fiscal, logistic and operational challenges in ensuring supply chain reliability and availability of paediatric formulations, and enormous progress has been made in South Africa in a rapid time 33. Initiatives such as the Global Accelerator for Paediatric Formulations (GAP‐f) have the potential to support state services across the region 34.

In relation to healthcare experiences for adolescents in the clinic, again four factors were unexpectedly not significantly associated with retention in the multivariate models. The provision of sufficient information and adolescent trust in the confidentiality of their health data were not associated with retention, and nor were treatment buddies or monthly support groups. However, we note that very few participants in this sample (14%) attended any support group, and only 5% attended an adolescent‐specific support group, and so this finding may reflect challenges for adolescents in relating to adult‐focused support services 6. Two staff‐related factors were strongly associated with increased adolescent retention: participant perceptions of healthcare providers who had time to spend with adolescents and who were kind to adolescents were both associated with more than 2.5 times the odds of retention in care. It is unclear whether hurried appointments were due to health provider attitudes, administrative burden or to high patient load. This supports qualitative data from the adult literature of the importance of the relationship and engagement with healthcare providers 35. Within Sub‐Saharan Africa, increasing use of community health workers and peer supporters within clinic settings (e.g. by Pediatric Adolescent Treatment for Africa 36) may allow increased time for adolescents even in overburdened clinics. There is no known evidence of successful programmes to improve healthcare worker‐adolescent engagement in the context of HIV care in the region 37, although in the US provider training in adolescent health was associated with higher retention in care in a cross‐sectional study 12. This is clearly an important area for providing future support to healthcare workers and managers.

This study has a number of limitations. First, all measurements are cross‐sectional and therefore we cannot determine causality. Second, clinic files had low records of viral load testing, with a third of files reporting no viral load test in the previous two years. Viral failure rates amongst untested adolescents are not known, and may have led to an underestimate of viral failure rates overall. Due to the limited available viral load data, we used tests recorded during the two years preceding the study, which introduces problems of temporality. For future studies that are conducted in low‐resource settings such as this one, where viral load testing is rare and inconsistent, it may be of value to conduct independent viral load assessments. These limitations reflect some of the challenges of conducting research within real‐world public health services in Africa, outside high‐quality teaching hospitals and donor‐funded clinics 38. This study uses self‐reported clinic non‐attendance and ART non‐adherence, which risk recall and social desirability biases. However, this study, as well as a number of others, found correlations between self‐reported retention and detectable viral load 39, 40. Rates of literacy and schooling varied amongst the study participants, with 94% of adolescents enrolled in school but 40% reporting some extent of cognitive difficulties (mostly mild). In order to facilitate engagement, interviewers read questionnaires aloud to participants who struggled with literacy. The study was limited to examine associations between retention and potential health service protective factors. New evidence suggests important factors beyond the health system, for example family and dating violence 41, 42 and treatment self‐efficacy 28. Future studies could valuably explore potential interaction effects between health service factors, and between social, psychological and health factors. Finally, the sample of eligible adolescents was limited to those who had engaged with HIV care at least once in their lives. Therefore, the study may be underestimating overall vulnerability of adolescents living with HIV in these communities, by not being able to include those who had never tested or initiated ART, or those who had died prior to the study starting.

However, some of the strengths of this study derive from its ‘real‐world services’ sample. High inclusion rates and community tracing of all adolescents initiated on ART allowed inclusion of adolescents regardless of whether they were retained in healthcare or not. We note, however, that the 9.9% who were not included due to false addresses, severe cognitive delay or refusal to participate may have been especially vulnerable to low retention in care, and are important groups to attempt to understand in terms of relationships with health services.

## 5 Conclusions

Despite limitations, these findings are important for informing adolescent‐responsive HIV service provision. Together, they suggest the potential for an intervention package that focuses on financial and moral support for adolescents (fares to the clinic, accompaniment to health services), and organizational/infrastructure support to services (stock flow, provider time) and healthcare workers to improve provider–patient interaction.

They also suggest that economic and psychosocial services may be valuable in supporting health system use and treatment adherence 38, 43. Two ongoing randomized trials in South Africa and Uganda examine family‐based and economic strengthening approaches to adherence support, and successful pilots suggest likely positive impacts 44, 45. Recent qualitative research with adult HIV patients in Zambia finds that experiences of health systems interact closely with patient characteristics and the social settings in which they negotiate their ART use 46. It will be important to further identify how social and economic services can support health services to improve adolescent retention in HIV care. These findings also demonstrate the potential value of providing combinations of protective factors. Whilst each protective factor alone was associated with a small increase in retention, the combination of all five STACK factors was associated with a rise of 66% in adolescent retention in care. By strengthening existing services and capacities within government health systems and communities, we have the potential to stack the odds for – not against – adolescents living with HIV.

## Competing interests

The authors declare that they have no competing interests.

## Authors’ contributions

LC and ET had key roles in designing the research study and managing the research data. MP and LS contributed to this research design and to the choice of measurement tools. LC, ET, MC, NB and MP were involved in the management of the field research. In particular, ET and NB oversaw the clinic data collection and management, and helped resolve complex clinic data issues. LC took the lead in conceptualizing the paper and writing it up. MP, LC and MO conducted the analysis. ET contributed to writing up the methods section. MC contributed to the literature reviews and provided conceptual input for the introduction and discussion sections. All authors approved the final version of the paper.
